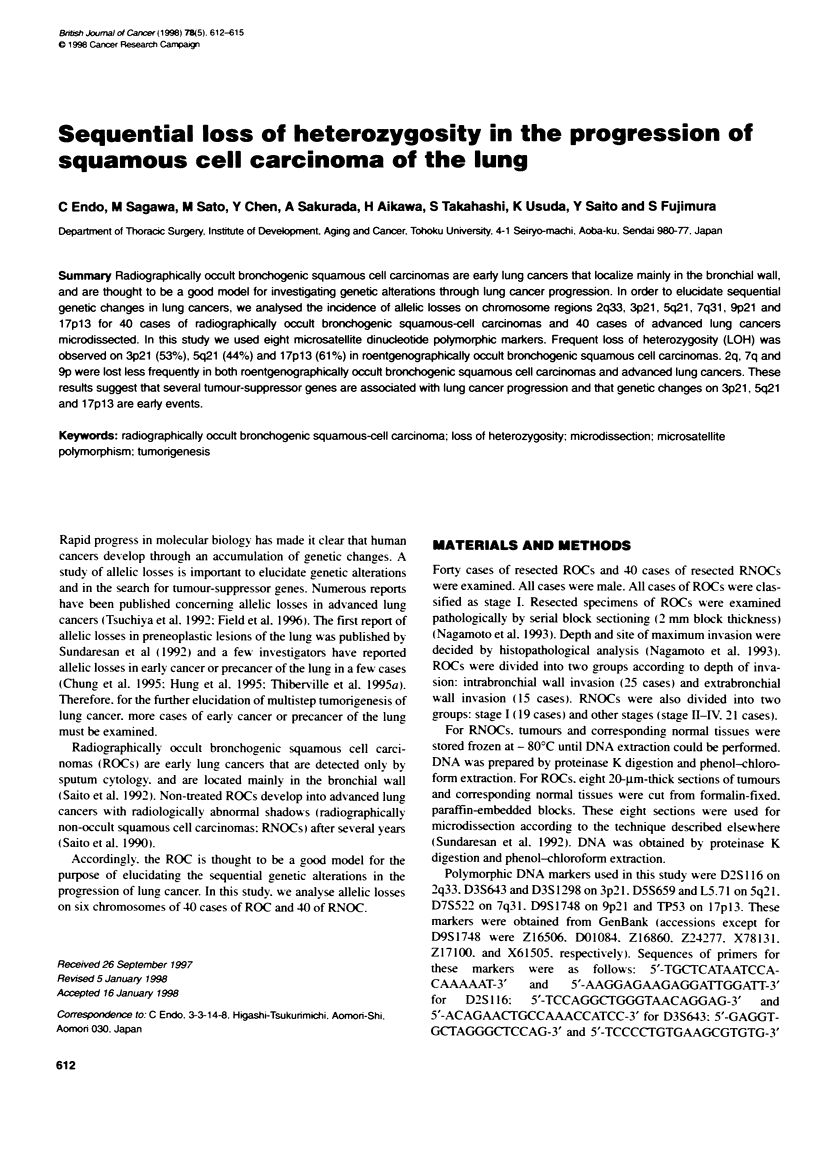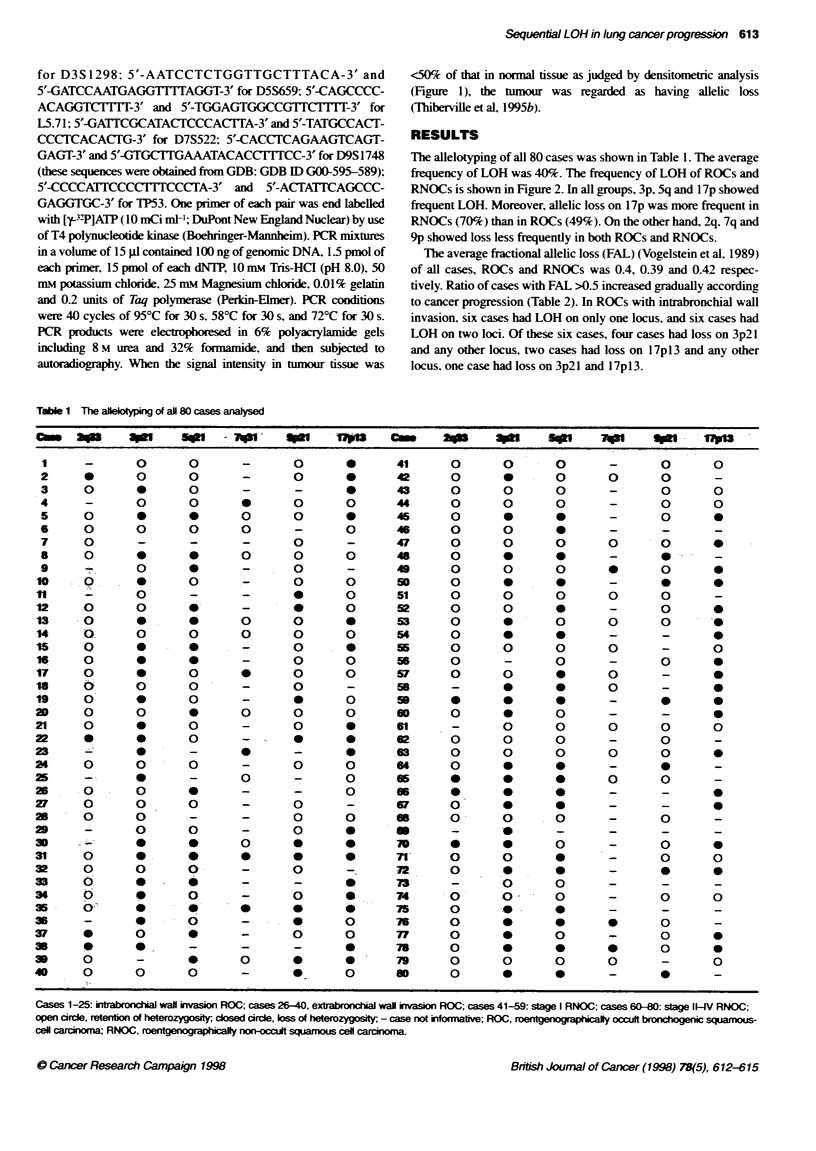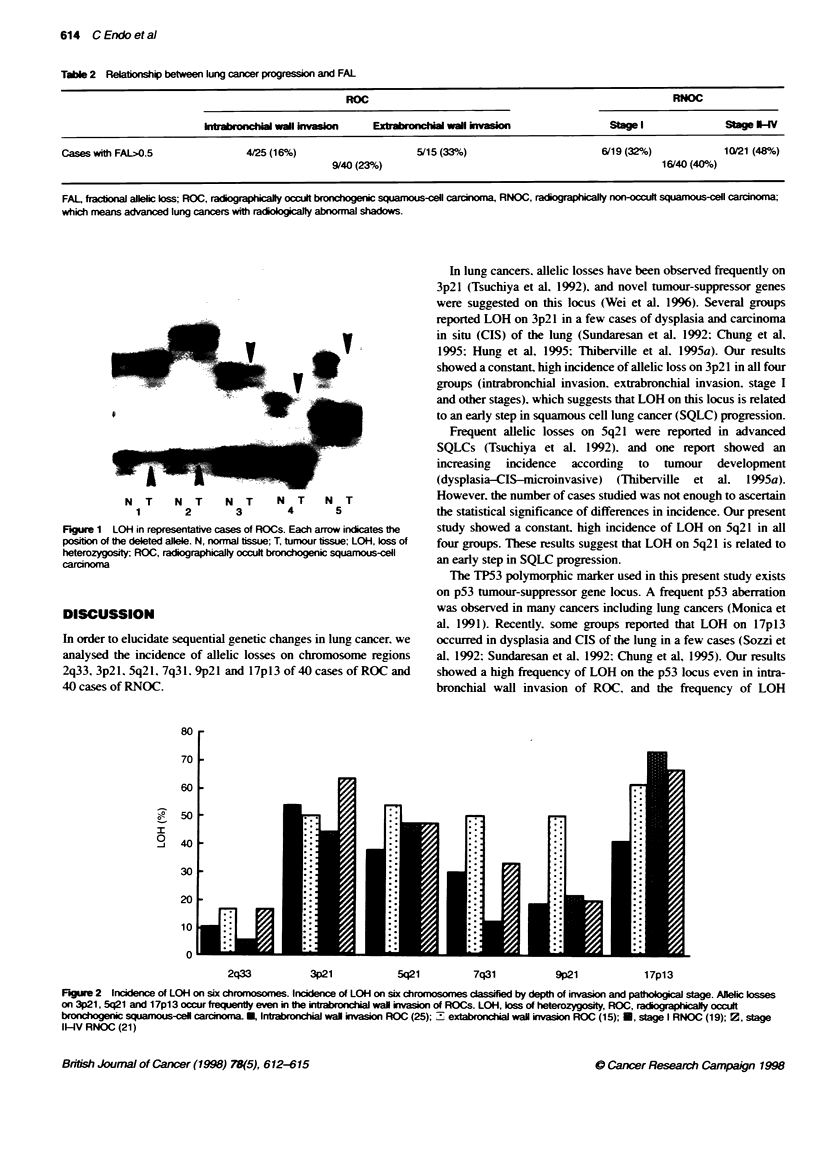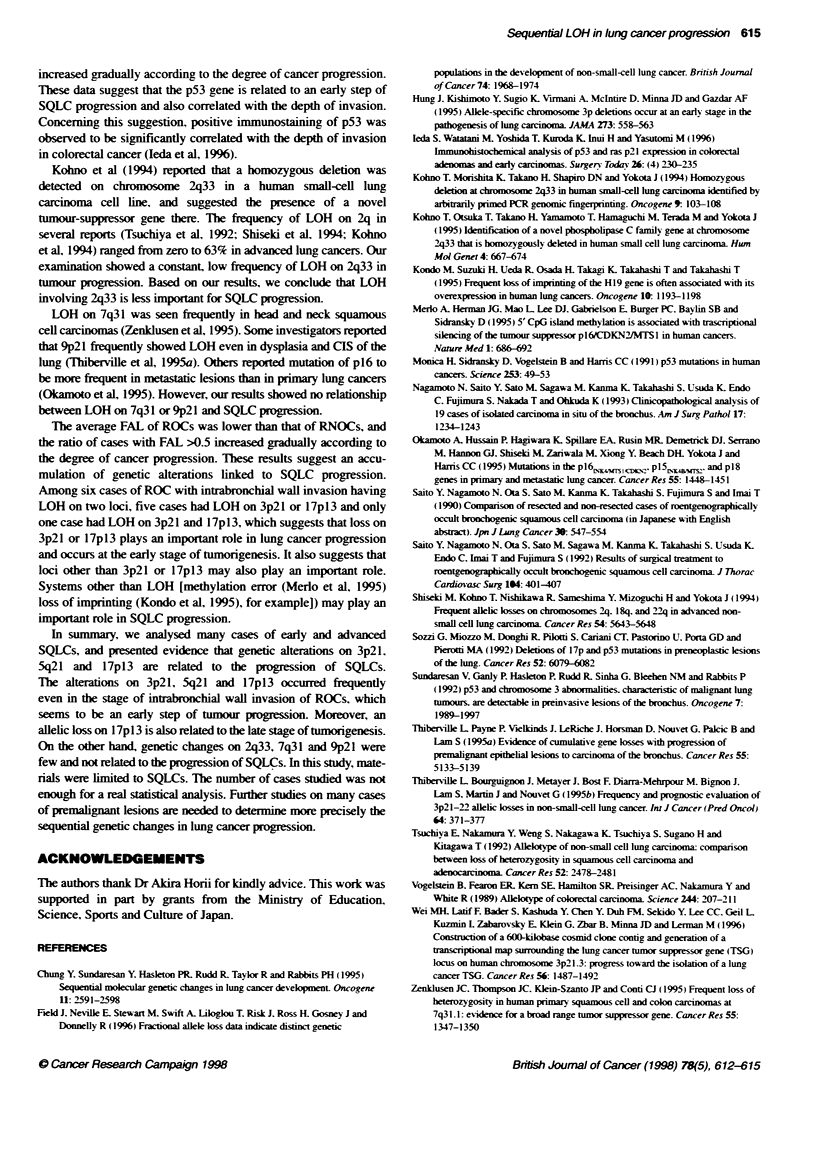# Sequential loss of heterozygosity in the progression of squamous cell carcinoma of the lung.

**DOI:** 10.1038/bjc.1998.549

**Published:** 1998-09

**Authors:** C. Endo, M. Sagawa, M. Sato, Y. Chen, A. Sakurada, H. Aikawa, S. Takahashi, K. Usuda, Y. Saito, S. Fujimura

**Affiliations:** Department of Thoracic Surgery, Institute of Development, Aging and Cancer, Tohoku University, Sendai, Japan.

## Abstract

**Images:**


*British Journal of Cancer* (1998) 78(5), 612–615
© 1998 Cancer Research Campaign

### Sequential loss of heterozygosity in the progression of squamous cell carcinoma of the lung

C Endo, M Sagawa, M Sato, Y Chen, A Sakurada, H Aikawa, S Takahashi, K Usuda, Y Saito and S Fujimura

Department of Thoracic Surgery, Institute of Development, Aging and Cancer, Tohoku University, 4-1 Seiryo-machi, Aoba-ku, Sendai 980-77, Japan

**Summary** Radiographically occult bronchogenic squamous cell carcinomas are early lung cancers that localize mainly in the bronchial wall, and are thought to be a good model for investigating genetic alterations through lung cancer progression. In order to elucidate sequential genetic changes in lung cancers, we analysed the incidence of allelic losses on chromosome regions 2q33, 3p21, 5q21, 7q31, 9p21 and 17p13 for 40 cases of radiographically occult bronchogenic squamous-cell carcinomas and 40 cases of advanced lung cancers microdissected. In this study we used eight microsatellite dinucleotide polymorphic markers. Frequent loss of heterozygosity (LOH) was observed on 3p21 (53%), 5q21 (44%) and 17p13 (61%) in roentgenographically occult bronchogenic squamous cell carcinomas. 2q, 7q and 9p were lost less frequently in both roentgenographically occult bronchogenic squamous cell carcinomas and advanced lung cancers. These results suggest that several tumour-suppressor genes are associated with lung cancer progression and that genetic changes on 3p21, 5q21 and 17p13 are early events.

**Keywords:** radiographically occult bronchogenic squamous-cell carcinoma; loss of heterozygosity; microdissection; microsatellite polymorphism; tumorigenesis

Rapid progress in molecular biology has made it clear that human cancers develop through an accumulation of genetic changes. A study of allelic losses is important to elucidate genetic alterations and in the search for tumour-suppressor genes. Numerous reports have been published concerning allelic losses in advanced lung cancers (Tsuchiya et al, 1992; Field et al, 1996). The first report of allelic losses in preneoplastic lesions of the lung was published by Sundaresan et al (1992) and a few investigators have reported allelic losses in early cancer or precancer of the lung in a few cases (Chung et al, 1995; Hung et al, 1995; Thiberville et al, 1995*a*). Therefore, for the further elucidation of multistep tumorigenesis of lung cancer, more cases of early cancer or precancer of the lung must be examined.

Radiographically occult bronchogenic squamous cell carcinomas (ROCs) are early lung cancers that are detected only by sputum cytology, and are located mainly in the bronchial wall (Saito et al, 1992). Non-treated ROCs develop into advanced lung cancers with radiologically abnormal shadows (radiographically non-occult squamous cell carcinomas; RNOCs) after several years (Saito et al, 1990).

Accordingly, the ROC is thought to be a good model for the purpose of elucidating the sequential genetic alterations in the progression of lung cancer. In this study, we analyse allelic losses on six chromosomes of 40 cases of ROC and 40 of RNOC.

*Received 26 September 1997*
*Revised 5 January 1998*
*Accepted 16 January 1998*

*Correspondence to:* C Endo, 3-3-14-8, Higashi-Tsukurimichi, Aomori-Shi, Aomori 030, Japan

### MATERIALS AND METHODS

Forty cases of resected ROCs and 40 cases of resected RNOCs were examined. All cases were male. All cases of ROCs were classified as stage I. Resected specimens of ROCs were examined pathologically by serial block sectioning (2 mm block thickness) (Nagamoto et al, 1993). Depth and site of maximum invasion were decided by histopathological analysis (Nagamoto et al, 1993). ROCs were divided into two groups according to depth of invasion: intrabronchial wall invasion (25 cases) and extrabronchial wall invasion (15 cases). RNOCs were also divided into two groups: stage I (19 cases) and other stages (stage II–IV, 21 cases).

For RNOCs, tumours and corresponding normal tissues were stored frozen at − 80°C until DNA extraction could be performed. DNA was prepared by proteinase K digestion and phenol–chloroform extraction. For ROCs, eight 20-μm-thick sections of tumours and corresponding normal tissues were cut from formalin-fixed, paraffin-embedded blocks. These eight sections were used for microdissection according to the technique described elsewhere (Sundaresan et al, 1992). DNA was obtained by proteinase K digestion and phenol–chloroform extraction.

Polymorphic DNA markers used in this study were D2S116 on 2q33, D3S643 and D3S1298 on 3p21, D5S659 and L5.71 on 5q21, D7S522 on 7q31, D9S1748 on 9p21 and TP53 on 17p13. These markers were obtained from GenBank (accessions except for D9S1748 were Z16506, D01084, Z16860, Z24277, X78131, Z17100, and X61505, respectively). Sequences of primers for these markers were as follows: 5′-TGCTCATAATCCA-CAAAAAT-3′ and 5′-AAGGAGAAGAGGATTGGATT-3′ for D2S116; 5′-TCCAGGCTGGGTAACAGGAG-3′ and 5′-ACAGAACTGCCAAACCATCC-3′ for D3S643; 5′-GAGGT-GCTAGGGCTCCAG-3′ and 5′-TCCCCTGTGAAGCGTGTG-3′

for D3S1298; 5′-AATCCTCTGGTTGCTTTACA-3′ and 5′-GATCCAATGAGGTTTTAGGT-3′ for D5S659; 5′-CAGCCCC-ACAGGTCTTTT-3′ and 5′-TGGAGTGGCCGTTCTTTT-3′ for L5.71; 5′-GATTCGCATACTCCCACTTA-3′ and 5′-TATGCCACT-CCCTCACACTG-3′ for D7S522; 5′-CACCTCAGAAGTCAGT-GAGT-3′ and 5′-GTGCTTGAAATACACCTTTCC-3′ for D9S1748 (these sequences were obtained from GDB: GDB ID G00-595–589); 5′-CCCCATTCCCCTTTCCCTA-3′ and 5′-ACTATTCAGCCC-GAGGTGC-3′ for TP53. One primer of each pair was end labelled with [$\gamma$-$^{32}$P]ATP (10 mCi ml$^{-1}$; DuPont New England Nuclear) by use of T4 polynucleotide kinase (Boehringer-Mannheim). PCR mixtures in a volume of 15 µl contained 100 ng of genomic DNA, 1.5 pmol of each primer, 15 pmol of each dNTP, 10 mM Tris-HCl (pH 8.0), 50 mM potassium chloride, 25 mM Magnesium chloride, 0.01% gelatin and 0.2 units of *Taq* polymerase (Perkin-Elmer). PCR conditions were 40 cycles of 95°C for 30 s, 58°C for 30 s, and 72°C for 30 s. PCR products were electrophoresed in 6% polyacrylamide gels including 8 M urea and 32% formamide, and then subjected to autoradiography. When the signal intensity in tumour tissue was <50% of that in normal tissue as judged by densitometric analysis (Figure 1), the tumour was regarded as having allelic loss (Thiberville et al, 1995*b*).

### RESULTS

The allelotyping of all 80 cases was shown in Table 1. The average frequency of LOH was 40%. The frequency of LOH of ROCs and RNOCs is shown in Figure 2. In all groups, 3p, 5q and 17p showed frequent LOH. Moreover, allelic loss on 17p was more frequent in RNOCs (70%) than in ROCs (49%). On the other hand, 2q, 7q and 9p showed loss less frequently in both ROCs and RNOCs.

The average fractional allelic loss (FAL) (Vogelstein et al, 1989) of all cases, ROCs and RNOCs was 0.4, 0.39 and 0.42 respectively. Ratio of cases with FAL >0.5 increased gradually according to cancer progression (Table 2). In ROCs with intrabronchial wall invasion, six cases had LOH on only one locus, and six cases had LOH on two loci. Of these six cases, four cases had loss on 3p21 and any other locus, two cases had loss on 17p13 and any other locus, one case had loss on 3p21 and 17p13.

**Table 1** The allelotyping of all 80 cases analysed

| Case | 2q33 | 3p21 | 5q21 | 7q31 | 9p21 | 17p13 | Case | 2q33 | 3p21 | 5q21 | 7q31 | 9p21 | 17p13 |
|---|---|---|---|---|---|---|---|---|---|---|---|---|---|
| 1 | – | ○ | ○ | – | ○ | ● | 41 | ○ | ○ | ○ | – | ○ | ○ |
| 2 | ● | ○ | ○ | – | ○ | ● | 42 | ○ | ● | ○ | ○ | ○ | – |
| 3 | ○ | ● | ○ | – | – | ● | 43 | ○ | ○ | ○ | – | ○ | ○ |
| 4 | – | ○ | ○ | ● | ○ | ○ | 44 | ○ | ○ | ○ | – | ○ | ○ |
| 5 | ○ | ● | ● | ○ | ○ | ● | 45 | ○ | ● | ● | – | ○ | ● |
| 6 | ○ | ○ | ○ | ○ | – | ○ | 46 | ○ | ○ | ● | – | – | – |
| 7 | ○ | – | – | – | ○ | – | 47 | ○ | ○ | ○ | ○ | ○ | ● |
| 8 | ○ | ● | ● | ○ | ○ | ○ | 48 | ○ | ● | ● | – | ● | – |
| 9 | – | ○ | ● | – | ○ | – | 49 | ○ | ○ | ○ | ● | ○ | ● |
| 10 | ○ | ● | ○ | – | ○ | ○ | 50 | ○ | ● | ● | – | ● | ● |
| 11 | – | ○ | – | – | ● | ○ | 51 | ○ | ○ | ○ | ○ | ○ | – |
| 12 | ○ | ○ | ● | – | ● | ○ | 52 | ○ | ○ | ● | – | ○ | ● |
| 13 | ○ | ● | ● | ○ | ○ | ● | 53 | ○ | ● | ○ | ○ | ○ | ● |
| 14 | ○ | ○ | ○ | ○ | ○ | ○ | 54 | ○ | ● | ● | – | – | ● |
| 15 | ○ | ● | ● | – | ○ | ● | 55 | ○ | ○ | ○ | ○ | – | ○ |
| 16 | ○ | ● | ● | – | ○ | ○ | 56 | ○ | – | ○ | – | ○ | ● |
| 17 | ○ | ● | ○ | ● | ○ | ○ | 57 | ○ | ○ | ● | ○ | – | ● |
| 18 | ○ | ○ | ○ | – | ○ | – | 58 | – | ● | ● | ○ | – | ● |
| 19 | ○ | ● | ○ | – | ● | ○ | 59 | ● | ● | ● | – | ● | ● |
| 20 | ○ | ○ | ● | ○ | ○ | ○ | 60 | ○ | ● | ○ | – | – | ● |
| 21 | ○ | ● | ○ | – | ○ | ● | 61 | – | ○ | ○ | ○ | ○ | ○ |
| 22 | ● | ● | ○ | – | ● | ● | 62 | ○ | ○ | ○ | – | ○ | – |
| 23 | – | ● | – | ● | – | ● | 63 | ○ | ○ | ○ | ○ | ○ | ● |
| 24 | ○ | ○ | ○ | – | ○ | ○ | 64 | ○ | ● | ● | – | ● | – |
| 25 | – | ● | – | ○ | – | ○ | 65 | ● | ● | ● | ○ | ○ | – |
| 26 | ○ | ○ | ● | – | – | ○ | 66 | ● | ● | ● | – | – | ● |
| 27 | ○ | ○ | ○ | – | ○ | – | 67 | ○ | ● | ● | – | – | ● |
| 28 | ○ | ○ | – | – | ○ | ○ | 68 | ○ | ○ | ○ | – | ○ | – |
| 29 | – | ○ | ○ | – | ○ | ● | 69 | – | ● | – | – | – | – |
| 30 | – | ● | ● | ○ | ● | ● | 70 | ● | ● | ○ | – | ○ | ● |
| 31 | ○ | ● | ● | ● | ● | ● | 71 | ○ | ○ | ● | – | ○ | ○ |
| 32 | ○ | ○ | ○ | – | ○ | – | 72 | ○ | ● | ● | – | ● | ● |
| 33 | ○ | ● | ● | – | – | ● | 73 | – | ○ | ○ | – | – | – |
| 34 | ○ | ● | ○ | – | ○ | ● | 74 | ○ | ○ | ○ | – | ○ | ○ |
| 35 | ○ | ● | ● | ● | ● | ● | 75 | ○ | ● | ● | – | – | – |
| 36 | – | ● | ○ | – | ● | ○ | 76 | ○ | ● | ● | ● | ○ | – |
| 37 | ● | ○ | ● | – | ○ | ○ | 77 | ○ | ● | ○ | – | ○ | ● |
| 38 | ● | ● | – | – | – | ● | 78 | ○ | ● | ● | ● | ○ | ● |
| 39 | ○ | – | ● | ○ | ● | ● | 79 | ○ | ○ | ○ | ○ | – | ○ |
| 40 | ○ | ○ | ○ | – | ● | ○ | 80 | ○ | ● | ● | – | ● | – |

Cases 1–25: intrabronchial wall invasion ROC; cases 26–40, extrabronchial wall invasion ROC; cases 41–59: stage I RNOC; cases 60–80: stage II–IV RNOC; open circle, retention of heterozygosity; closed circle, loss of heterozygosity; – case not informative; ROC, roentgenographically occult bronchogenic squamous-cell carcinoma; RNOC, roentgenographically non-occult squamous cell carcinoma.

© Cancer Research Campaign 1998

Table 2 Relationship between lung cancer progression and FAL

| | ROC | | RNOC | |
|---|---|---|---|---|
| | Intrabronchial wall invasion | Extrabronchial wall invasion | Stage I | Stage II–IV |
| Cases with FAL>0.5 | 4/25 (16%) | 5/15 (33%) | 6/19 (32%) | 10/21 (48%) |
| | 9/40 (23%) | | 16/40 (40%) | |

FAL, fractional allelic loss; ROC, radiographically occult bronchogenic squamous-cell carcinoma, RNOC, radiographically non-occult squamous-cell carcinoma; which means advanced lung cancers with radiologically abnormal shadows.

Figure 1 LOH in representative cases of ROCs. Each arrow indicates the position of the deleted allele. N, normal tissue; T, tumour tissue; LOH, loss of heterozygosity; ROC, radiographically occult bronchogenic squamous-cell carcinoma

### DISCUSSION

In order to elucidate sequential genetic changes in lung cancer, we analysed the incidence of allelic losses on chromosome regions 2q33, 3p21, 5q21, 7q31, 9p21 and 17p13 of 40 cases of ROC and 40 cases of RNOC.

In lung cancers, allelic losses have been observed frequently on 3p21 (Tsuchiya et al, 1992), and novel tumour-suppressor genes were suggested on this locus (Wei et al, 1996). Several groups reported LOH on 3p21 in a few cases of dysplasia and carcinoma in situ (CIS) of the lung (Sundaresan et al, 1992; Chung et al, 1995; Hung et al, 1995; Thiberville et al, 1995*a*). Our results showed a constant, high incidence of allelic loss on 3p21 in all four groups (intrabronchial invasion, extrabronchial invasion, stage I and other stages), which suggests that LOH on this locus is related to an early step in squamous cell lung cancer (SQLC) progression.

Frequent allelic losses on 5q21 were reported in advanced SQLCs (Tsuchiya et al, 1992), and one report showed an increasing incidence according to tumour development (dysplasia–CIS–microinvasive) (Thiberville et al, 1995*a*). However, the number of cases studied was not enough to ascertain the statistical significance of differences in incidence. Our present study showed a constant, high incidence of LOH on 5q21 in all four groups. These results suggest that LOH on 5q21 is related to an early step in SQLC progression.

The TP53 polymorphic marker used in this present study exists on p53 tumour-suppressor gene locus. A frequent p53 aberration was observed in many cancers including lung cancers (Monica et al, 1991). Recently, some groups reported that LOH on 17p13 occurred in dysplasia and CIS of the lung in a few cases (Sozzi et al, 1992; Sundaresan et al, 1992; Chung et al, 1995). Our results showed a high frequency of LOH on the p53 locus even in intrabronchial wall invasion of ROC, and the frequency of LOH

Figure 2 Incidence of LOH on six chromosomes. Incidence of LOH on six chromosomes classified by depth of invasion and pathological stage. Allelic losses on 3p21, 5q21 and 17p13 occur frequently even in the intrabronchial wall invasion of ROCs. LOH, loss of heterozygosity, ROC, radiographically occult bronchogenic squamous-cell carcinoma. ■, Intrabronchial wall invasion ROC (25); ⬚ extrabronchial wall invasion ROC (15); ▩, stage I RNOC (19); ▨, stage II–IV RNOC (21)

 © Cancer Research Campaign 1998

increased gradually according to the degree of cancer progression. These data suggest that the p53 gene is related to an early step of SQLC progression and also correlated with the depth of invasion. Concerning this suggestion, positive immunostaining of p53 was observed to be significantly correlated with the depth of invasion in colorectal cancer (Ieda et al, 1996).

Kohno et al (1994) reported that a homozygous deletion was detected on chromosome 2q33 in a human small-cell lung carcinoma cell line, and suggested the presence of a novel tumour-suppressor gene there. The frequency of LOH on 2q in several reports (Tsuchiya et al, 1992; Shiseki et al, 1994; Kohno et al, 1994) ranged from zero to 63% in advanced lung cancers. Our examination showed a constant, low frequency of LOH on 2q33 in tumour progression. Based on our results, we conclude that LOH involving 2q33 is less important for SQLC progression.

LOH on 7q31 was seen frequently in head and neck squamous cell carcinomas (Zenklusen et al, 1995). Some investigators reported that 9p21 frequently showed LOH even in dysplasia and CIS of the lung (Thiberville et al, 1995*a*). Others reported mutation of p16 to be more frequent in metastatic lesions than in primary lung cancers (Okamoto et al, 1995). However, our results showed no relationship between LOH on 7q31 or 9p21 and SQLC progression.

The average FAL of ROCs was lower than that of RNOCs, and the ratio of cases with FAL >0.5 increased gradually according to the degree of cancer progression. These results suggest an accumulation of genetic alterations linked to SQLC progression. Among six cases of ROC with intrabronchial wall invasion having LOH on two loci, five cases had LOH on 3p21 or 17p13 and only one case had LOH on 3p21 and 17p13, which suggests that loss on 3p21 or 17p13 plays an important role in lung cancer progression and occurs at the early stage of tumorigenesis. It also suggests that loci other than 3p21 or 17p13 may also play an important role. Systems other than LOH [methylation error (Merlo et al, 1995) loss of imprinting (Kondo et al, 1995), for example]) may play an important role in SQLC progression.

In summary, we analysed many cases of early and advanced SQLCs, and presented evidence that genetic alterations on 3p21, 5q21 and 17p13 are related to the progression of SQLCs. The alterations on 3p21, 5q21 and 17p13 occurred frequently even in the stage of intrabronchial wall invasion of ROCs, which seems to be an early step of tumour progression. Moreover, an allelic loss on 17p13 is also related to the late stage of tumorigenesis. On the other hand, genetic changes on 2q33, 7q31 and 9p21 were few and not related to the progression of SQLCs. In this study, materials were limited to SQLCs. The number of cases studied was not enough for a real statistical analysis. Further studies on many cases of premalignant lesions are needed to determine more precisely the sequential genetic changes in lung cancer progression.

### ACKNOWLEDGEMENTS

The authors thank Dr Akira Horii for kindly advice. This work was supported in part by grants from the Ministry of Education, Science, Sports and Culture of Japan.

### REFERENCES

Chung Y, Sundaresan Y, Hasleton PR, Rudd R, Taylor R and Rabbits PH (1995) Sequential molecular genetic changes in lung cancer development. *Oncogene* **11**: 2591–2598

Field J, Neville E, Stewart M, Swift A, Liloglou T, Risk J, Ross H, Gosney J and Donnelly R (1996) Fractional allele loss data indicate distinct genetic populations in the development of non-small-cell lung cancer. *British Journal of Cancer* **74**: 1968–1974

Hung J, Kishimoto Y, Sugio K, Virmani A, McIntire D, Minna JD and Gazdar AF (1995) Allele-specific chromosome 3p deletions occur at an early stage in the pathogenesis of lung carcinoma. *JAMA* **273**: 558–563

Ieda S, Watatani M, Yoshida T, Kuroda K, Inui H and Yasutomi M (1996) Immunohistochemical analysis of p53 and ras p21 expression in colorectal adenomas and early carcinomas. *Surgery Today* **26**: (4) 230–235

Kohno T, Morishita K, Takano H, Shapiro DN and Yokota J (1994) Homozygous deletion at chromosome 2q33 in human small-cell lung carcinoma identified by arbitrarily primed PCR genomic fingerprinting. *Oncogene* **9**: 103–108

Kohno T, Otsuka T, Takano H, Yamamoto T, Hamaguchi M, Terada M and Yokota J (1995) Identification of a novel phospholipase C family gene at chromosome 2q33 that is homozygously deleted in human small cell lung carcinoma. *Hum Mol Genet* **4**: 667–674

Kondo M, Suzuki H, Ueda R, Osada H, Takagi K, Takahashi T and Takahashi T (1995) Frequent loss of imprinting of the H19 gene is often associated with its overexpression in human lung cancers. *Oncogene* **10**: 1193–1198

Merlo A, Herman JG, Mao L, Lee DJ, Gabrielson E, Burger PC, Baylin SB and Sidransky D (1995) 5′ CpG island methylation is associated with trascriptional silencing of the tumour suppressor p16/CDKN2/MTS1 in human cancers. *Nature Med* **1**: 686–692

Monica H, Sidransky D, Vogelstein B and Harris CC (1991) p53 mutations in human cancers. *Science* **253**: 49–53

Nagamoto N, Saito Y, Sato M, Sagawa M, Kanma K, Takahashi S, Usuda K, Endo C, Fujimura S, Nakada T and Ohkuda K (1993) Clinicopathological analysis of 19 cases of isolated carcinoma in situ of the bronchus. *Am J Surg Pathol* **17**: 1234–1243

Okamoto A, Hussain P, Hagiwara K, Spillare EA, Rusin MR, Demetrick DJ, Serrano M, Hannon GJ, Shiseki M, Zariwala M, Xiong Y, Beach DH, Yokota J and Harris CC (1995) Mutations in the $p16_{INK4/MTS1/CDKN2}$, $p15_{INK4B/MTS2}$, and p18 genes in primary and metastatic lung cancer. *Cancer Res* **55**: 1448–1451

Saito Y, Nagamoto N, Ota S, Sato M, Kanma K, Takahashi S, Fujimura S and Imai T (1990) Comparison of resected and non-resected cases of roentgenographically occult bronchogenic squamous cell carcinoma (in Japanese with English abstract). *Jpn J Lung Cancer* **30**: 547–554

Saito Y, Nagamoto N, Ota S, Sato M, Sagawa M, Kanma K, Takahashi S, Usuda K, Endo C, Imai T and Fujimura S (1992) Results of surgical treatment to roentgenographically occult bronchogenic squamous cell carcinoma. *J Thorac Cardiovasc Surg* **104**: 401–407

Shiseki M, Kohno T, Nishikawa R, Sameshima Y, Mizoguchi H and Yokota J (1994) Frequent allelic losses on chromosomes 2q, 18q, and 22q in advanced non-small cell lung carcinoma. *Cancer Res* **54**: 5643–5648

Sozzi G, Miozzo M, Donghi R, Pilotti S, Cariani CT, Pastorino U, Porta GD and Pierotti MA (1992) Deletions of 17p and p53 mutations in preneoplastic lesions of the lung. *Cancer Res* **52**: 6079–6082

Sundaresan V, Ganly P, Hasleton P, Rudd R, Sinha G, Bleehen NM and Rabbits P (1992) p53 and chromosome 3 abnormalities, characteristic of malignant lung tumours, are detectable in preinvasive lesions of the bronchus. *Oncogene* **7**: 1989–1997

Thiberville L, Payne P, Vielkinds J, LeRiche J, Horsman D, Nouvet G, Palcic B and Lam S (1995*a*) Evidence of cumulative gene losses with progression of premalignant epithelial lesions to carcinoma of the bronchus. *Cancer Res* **55**: 5133–5139

Thiberville L, Bourguignon J, Metayer J, Bost F, Diarra-Mehrpour M, Bignon J, Lam S, Martin J and Nouvet G (1995*b*) Frequency and prognostic evaluation of 3p21–22 allelic losses in non-small-cell lung cancer. *Int J Cancer (Pred Oncol)* **64**: 371–377

Tsuchiya E, Nakamura Y, Weng S, Nakagawa K, Tsuchiya S, Sugano H and Kitagawa T (1992) Allelotype of non-small cell lung carcinoma: comparison between loss of heterozygosity in squamous cell carcinoma and adenocarcinoma. *Cancer Res* **52**: 2478–2481

Vogelstein B, Fearon ER, Kern SE, Hamilton SR, Preisinger AC, Nakamura Y and White R (1989) Allelotype of colorectal carcinoma. *Science* **244**: 207–211

Wei MH, Latif F, Bader S, Kashuda Y, Chen Y, Duh FM, Sekido Y, Lee CC, Geil L, Kuzmin I, Zabarovsky E, Klein G, Zbar B, Minna JD and Lerman M (1996) Construction of a 600-kilobase cosmid clone contig and generation of a transcriptional map surrounding the lung cancer tumor suppressor gene (TSG) locus on human chromosome 3p21.3: progress toward the isolation of a lung cancer TSG. *Cancer Res* **56**: 1487–1492

Zenklusen JC, Thompson JC, Klein-Szanto JP and Conti CJ (1995) Frequent loss of heterozygosity in human primary squamous cell and colon carcinomas at 7q31.1: evidence for a broad range tumor suppressor gene. *Cancer Res* **55**: 1347–1350

*© Cancer Research Campaign 1998*